# Supplementary material for: Habenula contributions to negative self-cognitions
Source: Nat Commun. 2025 May 7;16:4231. doi: 10.1038/s41467-025-59611-7 (PMC12059057; doi:10.1038/s41467-025-59611-7)
Supplement: Supplementary file 1 — Supplementary Information [file 41467_2025_59611_MOESM1_ESM.pdf]

Supplementary Information

Habenula contributions to negative self-cognitions

Kung, P-H., Greaves, MD., Guerrero-Hreins, E., Harrison, BJ., Davey, CG., Felmingham, KL., Carey, H., Sumithran, P., Brown, RM., Moffat, BA., Glarin, RK., Jamieson, AJ. & Steward, T.

Supplementary Methods **2**

Habenula resting-state functional connectivity (rsFC) 2

Timeseries extraction for the left-lateralised habenula effective connectivity model 2

Supplementary Results **3**

Endorsement of negative self-cognitions and perseverative thinking 3

Decisional bias to restructure or repeat self-cognitions 3

Activation patterns during the restructuring and repeating of self-cognitions 3

Supplementary Tables **4**

Supplementary Table 1. Participant demographics, Perseverative Thinking Questionnaire (PTQ), and endorsement of negative self-cognitions (CNBTQ) 4

Supplementary Table 2. Significant task-induced activation in the discovery sample 5

Supplementary Table 3. Effects of behavioural variables on habenula effective connectivity in the discovery sample 8

Supplementary Table 4. Bayesian model-averaged DCM parameters for endogenous and modulatory connections of the left-lateralised model 9

Supplementary Table 5. Significant task-induced activation in the replication sample 10

Supplementary Table 6. Bayesian model-averaged DCM parameters for endogenous and modulatory connections in the informed replication model 13

Supplementary Table 7. Bayesian model-averaged DCM parameters for endogenous and modulatory connections in the non-informed replication model 14

Supplementary Table 8. Participant demographics, Perseverative Thinking Questionnaire (PTQ), and endorsement of negative self-cognitions (CNBTQ) across the 5-fold validation subsamples 15

Supplementary Table 9. List of negative self-cognition statements in the cognitive restructuring paradigm 16

Supplementary Figures **17**

Supplementary Figure 1. Task-elicited neural activation in the discovery sample (*n* = 48) 17

Supplementary Figure 2. Task-elicited neural activation in the replication sample (*n* = 65) 18

Supplementary Figure 3. Overlapping activation across the discovery and replication datasets 19

Supplementary Figure 4. Intrinsic and modulatory connectivity comparison of the discovery and 5-fold validation models 20

Supplementary Figure 5. Overview of the habenula VOI preparation pipeline 21

Supplementary Figure 6. Participant negative self-cognition endorsement and choice bias during the cognitive restructuring task 22

Supplementary References **23**

Supplementary Methods

Habenula resting-state functional connectivity (rsFC)

Functional image acquisition

Resting-state fMRI was acquired from 50 participants in the discovery sample. During scanning, participants were instructed to remain in a relaxed wakeful state, while viewing a white fixation cross against a black background presented with a Microsoft PowerPoint 2021 slideshow. The resting-state sequence comprised a multi-band (factor = 6) and GRAPPA (R = 2) accelerated GE-EPI sequence in the steady state (TR = 800 ms; TE = 22.2 ms; pulse angle = 45°; field of view = 20.8 cm; acquisition matrix = 130 × 130-pixel; slice thickness = 1.6 mm, no gap; 84 interleaved axial slices). The sequence lasted just over 5 minutes in the single run, yielding a total of 400 whole-brain EPI volumes. Anatomical images used for this analysis were the same as those used for the task-based analyses.

Image pre-processing

Consistent with our task-based pipeline, image pre-processing for resting-state data was conducted using SPM12 (v7771; Wellcome Trust Centre for Neuroimaging, London) combined with the CONN toolbox, version 22a[1] within MATLAB 2023a (The MathWorks Inc., Natick, MA). The SPM pipeline included the following steps: realignment of the functional time series to the mean image for motion correction and resampling using 4th Degree B-Spline interpolation, co-registration of the anatomical images to their mean functional image, tissue segmentation using the unified segmentation plus DARTEL scheme, spatial normalisation to the ISBM European brain template using the DARTEL deformation fields, and smoothing of the resting-state functional image with a Gaussian kernel of 2 mm FWHM.

Standard denoising was conducted in CONN, including 6 motion parameters and their first-order derivatives, 5 CompCor noise components each from the DARTEL-segmented CSF and WM, the RETROICOR nuisance regressors generated via PhysIO toolbox[2], a binary array of motion outlier volumes identified by the CONN-based Artefact detection tool (ART), session effects and their first-order derivatives, as well as linear trends within the functional run. The time series was further band-pass filtered (0.008 - 0.09 Hz) to isolate the frequency of interest for rsFC analysis.

Seed-to-whole brain connectivity analysis

Seed-based connectivity maps were estimated in CONN for each individual using the subject-specific bilateral habenula mask as seed region. On the subject level, functional connectivity strength between the habenula seed and whole-brain voxels were represented as Fisher-transformed bivariate Pearson correlation coefficients using a weighted GLM. For the entire sample, whole-brain connectivity of the habenula was evaluated at each voxel using multivariate parametric statistics with random-effects across subjects. Results were thresholded and reported at whole-brain corrected $P_{FDR} < .05$, $K_E \geq 10$ voxels (Fig. 5d).

Timeseries extraction for the left-lateralised habenula effective connectivity model

For the exploratory left-lateralised model, we extracted representative time series from the left PCC, left hippocampus, and left pOFC following the same procedure as the right-lateralised model. Identical bilateral habenula ROIs were used, and all other network region ROIs were defined as 4 mm radius sphere centred around the contralateral (left-hemisphere) MNI coordinates derived from the subject-specific right-hemisphere ROI centre identified for the primary, right-lateralised DCM analysis (i.e., sign-flipping the x-coordinate of the right-hemisphere ROI centre). Volume-of-interest (VOI) from each of these regions were then calculated using SPM as the principal eigenvariate of all voxels within the respective ROI that showed meaningful activation for the task contrast ($p < .05$, uncorrected) at the single subject level. In the case when an ROI contains no voxel surpassing the preset threshold, the statistical threshold incrementally relaxed to $p < .5$ (uncorrected) to minimise data exclusion. This approach maximised topological symmetry across the lateralised models, ensuring that the right- and left-hemisphere ROIs reflect identical functional parcellations and allowing the direct examination of network functional laterality. VOIs were successfully extracted for all 45 participants of the discovery model sample and were included in the connectivity analysis.

Supplementary Results

Endorsement of negative self-cognitions and perseverative thinking

Participants in the discovery sample (n = 48) reported low levels of negative self-cognition endorsement both before (Mean = 2.26, SD = 0.94) and after (Mean = 2.24, SD = 0.95) the cognitive restructuring task. Although there were minor shifts in the participants' agreement with the negative self-cognition statements that were either restructured or repeated, these differences were not statistically significant (restructured: Mean = -0.09, t_{47} = -1.57, $P_{bonf.-corrected}$ = .246, 95% CI = [-0.19, 0.02]; repeated: Mean = 0.04, t_{47} = 0.61, $P_{bonf.-corrected}$ = 1.000, 95% CI = [-0.09, 0.16]). Likewise, participants in this sample reported only modest levels of perseverative thinking tendencies (Mean = 13.27, SD = 9.76).

Participants in the replication sample (n = 65) were comparable to the discovery group in male-female sex composition (χ^2 = 1.24, $P_{bonf.-corrected}$ = 1.000) but were significantly younger in age ($Mean_{difference}$ = 3.08, t_{111} = 3.45, $P_{bonf.-corrected}$ = .005, d = 4.69, 95% CI = [1.31, 4.85]). These participants reported similar levels of perseverative thinking (Mean = 18.83, SD = 13.80) as the discovery cohort ($Mean_{difference}$ = -5.56, U = 1215.50, $P_{bonf.-corrected}$ = .225). Of note, negative self-cognition endorsement in the replication group (Mean = 2.87, SD = 0.85) were significantly higher than the discovery sample ($Mean_{difference}$ = 0.61, t_{111} = 3.60, $P_{bonf.-corrected}$ < .001, d = 0.89, 95% CI = [0.27, 0.94]). There was also a significant reduction in their post-task endorsement of the self-cognition statements that were restructured (Mean = -0.54, t_{64} = -7.05, $P_{bonf.-corrected}$ < .001, d = 0.61, 95% CI = [-0.69, -0.38]), an effect that was significantly more pronounced than that of the discovery group ($Mean_{difference}$ = 0.45, U = 768.50, $P_{bonf.-corrected}$ < .001, r = 0.43, 95% CI = [0.27, 0.64]).

Decisional bias to restructure or repeat self-cognitions

Across both discovery and replication samples, the participants' choice to either restructure/challenge or repeat negative self-cognitions during the cognitive restructuring task appeared to depend on levels of negative self-cognition endorsement (Supplementary Fig. 6). Specifically, average negative self-cognition endorsement was significantly higher for statements that were repeated versus those that the participants restructured ($Mean_{difference}$ = -0.48, t_{98} = -6.70, $P_{bonf.-corrected}$ < .001, d = -0.67, 95% CI = [-0.62, -0.33]). Considering this, a decisional bias score was calculated for each participant as the difference in average endorsement ratings on statements that they chose to repeat versus restructure, with more positive values reflecting more pronounced choice tendencies to repeat statements that were more strongly endorsed. Participant bias score was significantly positively correlated with negative self-cognition endorsement (r = .33, $P_{bonf.-corrected}$ < .001, 95% CI = [0.14, 0.50]), suggesting that those with higher overall levels of negative self-cognition endorsement are more likely to show a stronger decisional bias to repeat more personally relevant statements as opposed to restructure them. This was further tested via k-mean clustering, where we broadly identified two sub-groups of participants who significantly differed on both their bias score and overall negative self-cognition endorsement (bias scores: $Mean_{difference}$ = -0.82, t_{97} = -7.02, $P_{bonf.-corrected}$ < .001, d = -1.41, 95% CI = [-1.05, -0.59]; endorsement ratings: $Mean_{difference}$ = -1.43, t_{97} = -11.30, $P_{bonf.-corrected}$ < .001, d = -2.28, 95% CI = [-1.69, -1.18]).

Activation patterns during the restructuring and repeating of self-cognitions

In order to ascertain the brain regions that are engaged in the processing of negative self-cognition, we used a conventional general linear model (GLM; n = 48) to examine the neural activation pattern during the restructuring and repeating of negative self-cognition statements. Cognitive restructuring, compared to repeating of the negative self-cognitions, elicited significant bilateral activation in regions of the frontostriatal cognitive control network, including the pre-supplementary motor area (pSMA) extending across the dorsomedial prefrontal cortex (dmPFC), the caudate, the thalamus, as well as the left dorsal to ventral lateral PFC (Fig. 2; Supplementary Fig. 1 & Table 2), consistent with previous reports[3, 4]. Repeating negative self-cognitions elicited increased activation in the right habenula, alongside key nodes of the default mode network, such as the bilateral inferior parietal lobule (IPL), posterior cingulate cortex (PCC), and the right ventromedial prefrontal cortex (vmPFC), right hippocampus and the right posterior orbitofrontal cortex (OFC).

Supplementary Tables

Supplementary Table 1. Participant demographics, Perseverative Thinking Questionnaire (PTQ), and endorsement of negative self-cognitions (CNBTQ)

	Discovery sample (n = 48)							Replication sample (n = 65)			
	**Male**				**Female**			**Male**		**Female**	
**Sex**	25 (52%)				23 (48%)			27 (41%)		38 (59%)	
	**Asian**	**Australian (non-ATSI)**	**British / European**	**North American**	**Central / South American**	**Middle Eastern**	**Multiple**	**Caucasian**	**Asian**	**Latinx**	**Mixed**
**Ethno-cultural groups**[a]	28	2	6	1	2	1	8	23	38	3	1
	**Mean**				**SD**			**Mean**		**SD**	
**Age, years**	25.83				5.17			22.75		4.30	
**Perseverative thinking questionnaire (PTQ)**	13.27				9.76			18.83		13.80	
**CNBTQ**[b] **(pre-task)**	2.26				0.94			2.87		0.85	

[a]Different information on ethno-cultural identity were obtained between the two projects from which the samples were derived.
[b]Challenging Negative Beliefs Task Questionnaire (CNBTQ) surveys participants' endorsement of the negative self-cognition statements (1 = Strongly Disagree; 7 = Strongly Agree) presented during the fMRI cognitive restructuring paradigm. This was completed before and after the MRI brain scan to assess changes in negative self-cognition endorsement after undergoing the cognitive restructuring task.
SD standard deviation.

Supplementary Table 2. Significant task-induced activation in the discovery sample

	MNI coordinates			Statistics	
	x	y	z	K_E	T value
**Challenge > Repeat**					
Supplementary motor area, L	-3	10	66	5632	8.89
Crus I of cerebellar hemisphere, R	43	-56	-32	4034	8.39
Caudate, L	-16	3	16	1545	7.84
Caudate, R[a]	19	3	13	1043	7.5
Middle frontal gyrus, L	-40	5	59	4824	6.83
Middle temporal gyrus, L	-51	-38	0	492	6.22
Crus I of cerebellar hemisphere, L	-45	-64	-27	490	6.18
IFG pars orbitalis, R	43	11	5	175	5.99
Lobule VIII of cerebellar hemisphere, L	-32	-50	-56	172	5.9
IFG pars orbitalis, R	48	21	-13	227	5.7
Superior temporal gyrus, R	50	-32	2	264	5.55
Middle occipital gyrus, R	38	-80	10	276	5.54
Locus coeruleus, L	-3	-34	-24	10	5.35
Superior frontal gyrus, dorsolateral, L	-11	53	35	469	4.89
Insula, L[a]	-34	3	3	20	4.84
Calcarine fissure and surrounding, L	-24	-56	6	68	4.82
Thalamus, Pulvinar medial, R[a]	0	-21	18	43	4.7
Crus I of cerebellar hemisphere, L	-32	-78	-27	13	4.66
Middle frontal gyrus, L	-34	46	18	361	4.64
Posterior cingulate gyrus, L[a]	-2	-32	10	22	4.62
Middle temporal gyrus, L	-58	-11	-14	98	4.61
Calcarine fissure and surrounding, L	-8	-93	10	140	4.58
Lobule IV, V of vermis[a]	-3	-53	2	154	4.47
Middle frontal gyrus, R	42	6	56	48	4.46
Lingual gyrus, R	30	-56	2	39	4.43
Red nucleus, L	-3	-18	-14	26	4.32
Thalamus, Mediodorsal medial magnocellular, L	-3	-24	-3	24	4.22
Middle frontal gyrus, L	-29	19	50	12	4.17
Lobule IX of cerebellar hemisphere, R	6	-58	-43	69	4.17
Middle frontal gyrus, R	32	40	27	22	4.14
Calcarine fissure and surrounding, R	16	-75	10	74	4.11
Thalamus, Medial Geniculate, L	-14	-24	-8	11	4.11
Inferior frontal gyrus, triangular part, R	50	29	2	13	4.03
Red nucleus, L[a]	-8	-21	-6	11	3.94
Calcarine fissure and surrounding, R	21	-64	11	13	3.9
Thalamus, Pulvinar medial, R[a]	2	-32	2	16	3.9
Lobule IX of cerebellar hemisphere, R	5	-51	-37	24	3.9
Middle occipital gyrus, R	34	-91	6	16	3.88
Superior frontal gyrus, dorsolateral, R	27	-2	59	10	3.75
Lobule IX of cerebellar hemisphere, L[a]	0	-43	-54	13	3.71
Angular gyrus, L	-30	-54	35	10	3.58
Lobule VIII of vermis, L[a]	0	-72	-48	10	3.52
**Repeat > Challenge**					
IFG pars orbitalis, L	-29	37	-10	545	7.72
Middle cingulate & paracingulate gyri, R	6	-26	46	15889	7.69
SupraMarginal gyrus, R	56	-40	40	8129	7.24
Inferior frontal gyrus, triangular part, R	48	46	-2	5317	6.94
Middle cingulate & paracingulate gyri, R	6	38	32	295	6.23

	MNI coordinates			Statistics	
	x	y	z	K_E	T value
Insula, R	42	-8	-10	693	5.8
Lingual gyrus, L	-5	-78	0	578	5.78
Inferior occipital gyrus, L	-42	-69	-5	836	5.58
Inferior frontal gyrus, opercular part, R	51	11	16	214	5.28
Superior parietal gyrus, L	-29	-62	66	281	5.23
Fusiform gyrus, L	-34	-21	-24	198	5.08
Precentral gyrus, L	-38	-8	50	21	5.05
Amygdala, L	-22	-3	-21	336	5.04
Thalamus, Pulvinar medial, R	18	-29	8	46	5.03
Lingual gyrus, L	-14	-38	-6	11	4.98
Unknown[a]	13	-24	-37	58	4.95
Superior frontal gyrus, dorsolateral, R	26	32	51	1463	4.9
Hippocampus, R	35	-26	-13	234	4.86
Precentral gyrus, R	30	-6	48	19	4.84
Thalamus, Pulvinar medial, L	-11	-30	10	42	4.82
Cuneus, L	-10	-64	26	344	4.76
Lingual gyrus, L	19	-83	-8	339	4.73
Middle occipital gyrus, L	-27	-83	30	657	4.73
Inferior occipital gyrus, L	-24	-96	-8	349	4.72
Inferior temporal gyrus, R	61	-38	-24	19	4.67
Inferior temporal gyrus, L	-56	-42	-18	40	4.64
Inferior temporal gyrus, R	38	-5	-38	16	4.54
Caudate, R	10	18	11	37	4.53
Lobule IV, V of cerebellar hemisphere, L[a]	-11	-27	-35	33	4.52
Lingual gyrus, L	-22	-67	-11	51	4.5
Hippocampus, R	24	-38	3	31	4.44
Caudate, L[a]	-2	21	8	10	4.34
Precentral gyrus, L[a]	-37	0	22	26	4.32
Middle temporal gyrus, L	-62	-58	0	23	4.32
Superior frontal gyrus, medial, L	-10	45	18	14	4.31
Fusiform gyrus, R	42	-35	-18	45	4.3
Temporal pole: middle temporal gyrus, R	35	24	-34	48	4.3
Amygdala, R	24	-2	-18	64	4.28
Medial orbital gyrus, L[a]	-11	61	-21	36	4.27
Postcentral gyrus, R	38	-32	67	26	4.26
Heschl's gyrus, R	56	-6	5	27	4.21
Superior frontal gyrus, dorsolateral, L	-30	43	42	18	4.15
Inferior temporal gyrus, R	53	-5	-35	15	4.14
Middle temporal gyrus, L	-66	-42	-11	25	4.13
Parahippocampal gyrus, R	24	0	-37	13	4.09
Inferior temporal gyrus, R	45	-42	-19	19	4.08
Crus II of cerebellar hemisphere, L	-42	-74	-40	32	4.06
Superior frontal gyrus, dorsolateral, R	21	-11	72	10	4.03
Thalamus, Mediodorsal medial magnocellular, R	3	-21	3	13	4.03
Unknown[a]	11	-14	-37	21	4.02
Superior occipital gyrus, L	-22	-66	32	15	3.98
Superior frontal gyrus, medial, L	-6	38	29	16	3.97
Amygdala, L[a]	-27	-2	-13	11	3.96
Precuneus, L	-13	-53	66	10	3.95
Hippocampus, L	-18	-37	6	10	3.94
Temporal pole: middle temporal gyrus, L	-26	10	-40	17	3.94
Caudate, R[a]	3	13	10	13	3.92

	MNI coordinates			Statistics	
	**x**	**y**	**z**	**K_E**	**T value**
Inferior temporal gyrus, L	-42	-46	-14	43	3.92
Fusiform gyrus, R	29	10	-46	18	3.9
Middle occipital gyrus, L	-38	-70	16	54	3.86
Cuneus, L	-5	-74	30	12	3.8
Precentral gyrus, R	61	2	32	21	3.78
Inferior temporal gyrus, L	-59	-34	-24	63	3.77
Caudate, L[a]	-3	10	18	14	3.73
Postcentral gyrus, R	32	-32	56	12	3.68
Middle frontal gyrus, L	-29	32	42	30	3.67
Precentral gyrus, L	-30	-10	48	10	3.66
Superior frontal gyrus, dorsolateral, R	21	24	42	26	3.64
Precentral gyrus, R	34	-27	70	15	3.62
Rolandic operculum, R	38	-30	18	12	3.58
Hippocampus, L	-27	-24	-13	13	3.57
Caudate, L	-3	10	-2	17	3.57
Unknown[a]	5	-24	-34	16	3.56
Superior frontal gyrus, medial, R	14	38	45	26	3.56
Superior temporal gyrus, R	45	-29	14	21	3.55
Superior frontal gyrus, medial, R	3	56	30	12	3.5
Temporal pole: superior temporal gyrus, R	29	5	-22	23	3.48
Middle occipital gyrus, L	-38	-86	18	14	3.48
Middle occipital gyrus, L	-42	-74	8	23	3.45
Precentral gyrus, R	30	-21	56	10	3.45
Cuneus, L[a]	-22	-54	26	25	3.44
Rolandic operculum, R	56	-11	10	29	3.44
Superior frontal gyrus, medial orbital, R	11	66	-8	11	3.43
Superior frontal gyrus, dorsolateral, R	24	11	51	14	3.42
Lingual gyrus, R	11	-37	3	11	3.4
Precentral gyrus, R	43	-10	54	30	3.38
Middle temporal gyrus, L	-40	-62	13	24	3.26
Superior frontal gyrus, dorsolateral, L	-30	58	-6	13	3.21
Middle cingulate & paracingulate gyri, R	2	2	32	18	3.05

Anatomic regions are defined with Automated Anatomical Labelling Atlas 3 (AAL3; local maxima labelling, 1mm voxel edge)[5]. Significant clusters are thresholded at $P_{FDR}<0.05$, $K_E \geq 10$ voxels. T values represent peak activation for the cluster.

[a]Cluster peak coordinates fall outside of the defined regions, hence, were labelled using the nearest available anatomic region using AAL3. Region labelled as “Unknown” if no applicable AAL3 label can be defined.

MNI Montreal Neurological Institute, *L* left, *R* right.

Supplementary Table 3. Effects of behavioural variables on habenula effective connectivity in the discovery sample

	CNBTQ			PTQ		
**Connection**	**Ep**	**Cp**	**PP**	**Ep**	**Cp**	**PP**
**Endogenous connections[a] (A-matrix)**						
Habenula → Habenula	< -0.01	< 0.0001	.00	< 0.01	< 0.0001	.00
Habenula → PCC	< -0.01	< 0.0001	.00	< 0.01	< 0.0001	.00
Habenula → Hippocampus	< -0.01	< 0.0001	.00	< 0.01	< 0.0001	.00
Habenula → pOFC	0.05	0.0007	.87	< 0.01	< 0.0001	.00
PCC → PCC	< 0.01	< 0.0001	.00	< 0.01	< 0.0001	.00
PCC → Habenula	< -0.01	< 0.0001	.00	< 0.01	< 0.0001	.00
Hippocampus → Hippocampus	< -0.01	< 0.0001	.00	< 0.01	< 0.0001	.00
Hippocampus → Habenula	< -0.01	< 0.0001	.00	< 0.01	< 0.0001	.00
pOFC → pOFC	-0.11	0.0041	.87	< 0.01	< 0.0001	.00
pOFC → Habenula	< 0.01	< 0.0001	.00	< 0.01	< 0.0001	.00
**Modulatory connections[b] (B-matrix)**						
**Challenge (CHAL)**						
Habenula → PCC	< -0.01	< 0.0001	.00	< -0.01	< 0.0001	.00
Habenula → Hippocampus	< -0.01	< 0.0001	.00	< 0.01	< 0.0001	.00
Habenula → pOFC	< 0.01	< 0.0001	.00	< 0.01	< 0.0001	.00
PCC → Habenula	< -0.01	< 0.0001	.00	< -0.01	< 0.0001	.00
Hippocampus → Habenula	< 0.01	< 0.0001	.00	< -0.01	< 0.0001	.00
pOFC → Habenula	< 0.01	< 0.0001	.00	< 0.01	< 0.0001	.00
**Repeat (REP)**						
Habenula → PCC	< 0.01	< 0.0001	.00	< -0.01	< 0.0001	.00
Habenula → Hippocampus	< 0.01	< 0.0001	.00	< -0.01	< 0.0001	.00
Habenula → pOFC	< -0.01	< 0.0001	.00	< 0.01	< 0.0001	.00
PCC → Habenula	< 0.01	< 0.0001	.00	< 0.01	< 0.0001	.00
Hippocampus → Habenula	-0.25	0.0339	.76	< -0.01	< 0.0001	.00
pOFC → Habenula	< 0.01	< 0.0001	.00	< 0.01	< 0.0001	.00

[a]Endogenous parameters reflect the average effective coupling between regions across experimental conditions (context-independent).
[b]Modulatory parameters reflect the changes in effective coupling between regions induced by cognitive reappraisal (content-dependent).
*Posterior probability (PP) exceeding .95 provides sufficient evidence for a non-zero group effect[6].
CNBTQ Challenging Negative Beliefs Questionnaire, *Cp* posterior covariance, *Ep* posterior expectation, *PCC* posterior cingulate cortex, *pOFC* posterior orbitofrontal cortex, *PP* posterior probability, *PTQ* Perseverative Thinking Questionnaire.

Supplementary Table 4. Bayesian model-averaged DCM parameters for endogenous and modulatory connections of the left-lateralised model

Connection	Ep	Cp	PP
**Endogenous connections[a] (A-matrix)**			
Habenula → Habenula	-0.42	0.0021	1.00*
Habenula → PCC	-0.12	0.0006	1.00*
Habenula → Hippocampus	-0.06	0.0010	.86
Habenula → pOFC	< 0.01	< 0.0001	.00
PCC → PCC	-0.21	0.0024	1.00*
PCC → Habenula	< -0.01	< 0.0001	.00
Hippocampus → Hippocampus	-0.19	0.0031	1.00*
Hippocampus → Habenula	< -0.01	< 0.0001	.00
pOFC → pOFC	-0.20	0.0024	1.00*
pOFC → Habenula	0.04	0.0013	.64
**Modulatory connections[b] (B-matrix)**			
**Challenge (CHAL)**			
Habenula → PCC	0.38	0.0067	1.00*
Habenula → Hippocampus	0.27	0.0044	1.00*
Habenula → pOFC	0.38	0.0046	1.00*
PCC → Habenula	0.56	0.0107	1.00*
Hippocampus → Habenula	< 0.01	< 0.0001	.00
pOFC → Habenula	< -0.01	< 0.0001	.00
**Repeat (REP)**			
Habenula → PCC	< 0.01	< 0.0001	.00
Habenula → Hippocampus	< 0.01	< 0.0001	.00
Habenula → pOFC	< -0.01	< 0.0001	.00
PCC → Habenula	< 0.01	< 0.0001	.00
Hippocampus → Habenula	< 0.01	< 0.0001	.00
pOFC → Habenula	< 0.01	< 0.0001	.00

[a]Endogenous parameters reflect the average effective coupling between regions across experimental conditions (context-independent).
[b]Modulatory parameters reflect the changes in effective coupling between regions induced by cognitive reappraisal (content-dependent).
*Posterior probability (PP) exceeding .95 provides sufficient evidence for a non-zero group effect[6].
Cp posterior covariance, *Ep* posterior expectation, *PCC* posterior cingulate cortex, *pOFC* posterior orbitofrontal cortex, *PP* posterior probability.

Supplementary Table 5. Significant task-induced activation in the replication sample

	MNI coordinates			Statistics	
	x	y	z	K_E	T value
**Challenge > Repeat**					
Supplementary motor area, L	-6	13	66	10342	10.84
Caudate, L	-16	6	14	12308	8.77
Lobule VI of cerebellar hemisphere, R	30	-53	-27	13660	8.36
Caudate, R	16	10	16	1505	7.3
Temporal pole: middle temporal gyrus, R	50	19	-27	416	6.86
Calcarine fissure and surrounding, L	-21	-64	8	32	5.89
Middle frontal gyrus, L	-32	48	19	517	5.6
Superior temporal gyrus, R	45	-32	0	337	5.58
Middle temporal gyrus, L	-51	-64	21	540	5.46
Substantia nigra, pars reticulata, R	13	-18	-14	709	5.35
Thalamus, Ventral lateral, R	18	-10	11	44	4.81
Thalamus, Pulvinar medial, R[a]	2	-29	8	24	4.42
Superior temporal gyrus, R	51	-16	-6	45	4.1
Crus I of cerebellar hemisphere, L	-48	-66	-24	13	4.06
Insula, L	-26	14	-16	15	4.04
Fusiform gyrus, L	-18	-42	-14	16	3.97
Lobule IV, V of cerebellar hemisphere, R	22	-30	-26	101	3.97
Lobule III of cerebellar hemisphere, R	16	-37	-29	12	3.94
Hippocampus, R[a]	32	-43	6	13	3.88
Temporal pole: middle temporal gyrus, R	38	10	-40	28	3.85
Lenticular nucleus, Pallidum, R	18	0	0	25	3.85
Cuneus, R	21	-80	46	49	3.81
Insula, R	35	19	3	21	3.79
Insula, R[a]	26	-30	24	14	3.77
Lingual gyrus, R[a]	8	-93	-18	20	3.73
Nucleus accumbens, R	10	16	-8	13	3.68
Olfactory cortex, L	0	22	-6	23	3.68
Lobule VIIB of cerebellar hemisphere, L	-6	-77	-45	17	3.67
Precuneus, L	-19	-50	6	16	3.63
Lobule VIII of cerebellar hemisphere, L	-34	-53	-51	29	3.61
Lobule IV, V of cerebellar hemisphere, L	-14	-37	-19	12	3.55
Hippocampus, R[a]	27	-35	14	13	3.55
Middle temporal gyrus, L[a]	-35	-54	16	16	3.5
Middle frontal gyrus, L	-30	40	29	17	3.48
Lingual gyrus, L	-16	-61	0	11	3.36
Caudate, R[a]	21	-14	16	12	3.34
SupraMarginal gyrus, L	-48	-45	24	22	3.33
Lobule IV, V of cerebellar hemisphere, L	-10	-38	-16	10	3.26
Calcarine fissure and surrounding, L[a]	-27	-58	8	15	3.22
Superior temporal gyrus, R	54	-6	-14	10	3.16
**Repeat > Challenge**					
SupraMarginal gyrus, R	58	-34	40	40113	9.61
Middle frontal gyrus, R	40	45	3	4918	7.84
Middle temporal gyrus, L	-53	-64	-5	2889	6.62
Superior frontal gyrus, medial, R	5	35	42	478	6.59
Insula, R	26	21	-19	236	5.77
Middle frontal gyrus, L	-29	37	-13	402	5.65
Lingual gyrus, R	18	-85	-3	1130	5.5
Amygdala, R	27	-2	-19	222	5.33

	MNI coordinates			Statistics	
	x	y	z	K_E	T value
Lobule VIII of cerebellar hemisphere, L	-27	-70	-50	453	5.26
Temporal pole: middle temporal gyrus, L[a]	-29	13	-46	50	5.1
Superior frontal gyrus, dorsolateral, R	21	11	56	305	4.97
Supplementary motor area, L	-10	-11	54	16	4.82
Lingual gyrus, L	-11	-77	-6	1334	4.82
Precentral gyrus, L	-18	-14	69	38	4.68
Middle frontal gyrus, L	-35	54	-6	105	4.67
Temporal pole: middle temporal gyrus, R	29	8	-42	31	4.59
Precentral gyrus, L	-19	-27	61	39	4.58
Middle temporal gyrus, L	-50	-34	-16	82	4.52
Inferior frontal gyrus, triangular part, L	-46	38	16	300	4.5
Anterior cingulate cortex, pregenual, R	11	43	8	54	4.39
Temporal pole: superior temporal gyrus, R	26	13	-35	20	4.27
Middle cingulate & paracingulate gyri, R	3	6	34	18	4.21
Thalamus, Pulvinar medial, L	-14	-27	3	35	4.18
Amygdala, L	-24	3	-18	29	4.16
Inferior temporal gyrus, L	-46	-13	-35	20	4.13
Thalamus, Pulvinar medial, L	16	-26	6	40	4.08
Parahippocampal gyrus, L	-16	-3	-21	33	4.05
Superior parietal gyrus, R	21	-53	72	29	4.02
Temporal pole: middle temporal gyrus, L	-27	8	-34	16	3.99
Middle frontal gyrus, L	-34	35	42	78	3.98
Fusiform gyrus, L	40	-18	-26	31	3.93
Caudate, L[a]	-24	-22	30	19	3.88
Fusiform gyrus, R[a]	38	-6	-27	24	3.87
Inferior temporal gyrus, L	-56	-19	-32	14	3.87
Middle frontal gyrus, R	48	22	34	57	3.86
Hippocampus, R	26	-34	-3	36	3.86
Superior frontal gyrus, dorsolateral, L[a]	-18	30	26	19	3.84
Insula, R	34	18	-19	27	3.82
Inferior temporal gyrus, R	51	-53	-21	19	3.78
Anterior orbital gyrus, L	-21	42	-14	46	3.76
Middle temporal gyrus, L[a]	-34	-64	16	31	3.73
Lobule IX of cerebellar hemisphere, R[a]	16	-38	-54	11	3.72
Parahippocampal gyrus, R[a]	13	-11	-37	12	3.69
Medial orbital gyrus, L	-10	58	-22	22	3.68
Crus I of cerebellar hemisphere, L	-26	-62	-35	15	3.68
Superior frontal gyrus, medial, L	-6	42	26	23	3.67
Middle cingulate & paracingulate gyri, L	-13	-43	42	12	3.65
Caudate, R[a]	3	5	6	31	3.65
Inferior temporal gyrus, R[a]	45	2	-30	12	3.57
Superior frontal gyrus, medial, L	-5	27	53	48	3.56
Lobule VI of cerebellar hemisphere, L	-24	-78	-21	30	3.56
Hippocampus, L	-21	-40	0	10	3.52
Superior frontal gyrus, medial, R[a]	3	46	54	38	3.52
Superior frontal gyrus, dorsolateral, R	16	-8	59	16	3.51
Crus I of cerebellar hemisphere, L	-43	-46	-43	19	3.49
Inferior temporal gyrus, L	-32	2	-42	11	3.49
Parahippocampal gyrus, L[a]	-11	-16	-34	15	3.48
Fusiform gyrus, R	35	-2	-38	30	3.48
Middle occipital gyrus, L	-34	-85	27	84	3.39
Superior frontal gyrus, dorsolateral, R	32	59	19	24	3.39
Thalamus, Pulvinar medial, L[a]	-13	-32	16	10	3.39

	MNI coordinates			Statistics	
	x	y	z	K_E	T value
Precentral gyrus, R	14	-22	75	34	3.37
Middle cingulate & paracingulate gyri, L	-3	0	40	17	3.35
Fusiform gyrus, L	-30	-37	-26	15	3.34
Superior frontal gyrus, dorsolateral, L	-16	51	-13	10	3.31
Paracentral lobule, L	-11	-37	74	10	3.2
Hippocampus, L	-32	-18	-22	13	3.14
Supplementary motor area, R[a]	19	-10	48	30	3.14
Superior frontal gyrus, medial, R	14	72	10	15	3.04

Anatomic regions are defined with Automated Anatomical Labelling Atlas 3 (AAL3; local maxima labelling, 1mm voxel edge)[5]. Significant clusters are thresholded at $P_{FDR}<0.05$, $K_E\geq10$ voxels. T values represent peak activation for the cluster.

[a]Cluster peak coordinates fall outside of the defined regions, hence, were labelled using the nearest available anatomic region using AAL3. Region labelled as "Unknown" if no applicable AAL3 label can be defined.

MNI Montreal Neurological Institute, *L* left, *R* right.

Supplementary Table 6. Bayesian model-averaged DCM parameters for endogenous and modulatory connections in the informed replication model

Connection	Ep	Cp	PP
**Endogenous connections[a] (A-matrix)**			
Habenula → Habenula	< 0.01	< 0.0001	.00
Habenula → PCC	-0.19	0.0005	1.00*
PCC → PCC	-0.43	0.0010	1.00*
PCC → Habenula	< 0.01	< 0.0001	.00
Hippocampus → Hippocampus	< 0.01	< 0.0001	.00
Hippocampus → Habenula	< 0.01	< 0.0001	.00
pOFC → pOFC	< -0.01	< 0.0001	.00
pOFC → Habenula	0.11	0.0015	.94
**Modulatory connections[b] (B-matrix)**			
**Challenge (CHAL)**			
Habenula → PCC	0.60	0.0770	.84
Habenula → pOFC	0.33	0.0037	1.00*
**Repeat (REP)**			
Habenula → PCC	0.37	0.0322	.84

[a]Endogenous parameters reflect the average effective coupling between regions across experimental conditions (context-independent).
[b]Modulatory parameters reflect the changes in effective coupling between regions induced by cognitive reappraisal (content-dependent).
*Posterior probability (PP) exceeding .95 provides sufficient evidence for a non-zero group effect[6].
Cp posterior covariance*,* *Ep* posterior expectation, *PCC* posterior cingulate cortex, *pOFC* posterior orbitofrontal cortex, *PP* posterior probability.

Supplementary Table 7. Bayesian model-averaged DCM parameters for endogenous and modulatory connections in the non-informed replication model

Connection	Ep	Cp	PP
**Endogenous connections[a] (A-matrix)**			
Habenula → Habenula	-0.39	0.0026	1.00*
Habenula → PCC	-0.16	0.0012	1.00*
Habenula → Hippocampus	-0.06	0.0014	.81
Habenula → pOFC	-0.06	0.0013	.84
PCC → PCC	-0.39	0.0017	1.00*
PCC → Habenula	< -0.01	< 0.0001	.00
Hippocampus → Hippocampus	-0.34	0.0021	1.00*
Hippocampus → Habenula	< -0.01	< 0.0001	.00
pOFC → pOFC	< -0.01	< 0.0001	.00
pOFC → Habenula	0.07	0.0019	.82
**Modulatory connections[b] (B-matrix)**			
**Challenge (CHAL)**			
Habenula → PCC	0.49	0.0213	.99*
Habenula → Hippocampus	0.16	0.0180	.68
Habenula → pOFC	0.38	0.0105	1.00*
PCC → Habenula	< -0.01	< 0.0001	.00
Hippocampus → Habenula	< -0.01	< 0.0001	.00
pOFC → Habenula	< -0.01	< 0.0001	.00
**Repeat (REP)**			
Habenula → PCC	0.29	0.0265	.85
Habenula → Hippocampus	< 0.01	< 0.0001	.00
Habenula → pOFC	0.26	0.0147	.92
PCC → Habenula	< -0.01	< 0.0001	.00
Hippocampus → Habenula	< -0.01	< 0.0001	.00
pOFC → Habenula	-0.17	0.0352	.56

[a]Endogenous parameters reflect the average effective coupling between regions across experimental conditions (context-independent).
[b]Modulatory parameters reflect the changes in effective coupling between regions induced by cognitive reappraisal (content-dependent).
*Posterior probability (PP) exceeding .95 provides sufficient evidence for a non-zero group effect[6].
Ep posterior expectation, *PCC* posterior cingulate cortex, *pOFC* posterior orbitofrontal cortex, *PP* posterior probability.

Supplementary Table 8. Participant demographics, Perseverative Thinking Questionnaire (PTQ), and endorsement of negative self-cognitions (CNBTQ) across the 5-fold validation subsamples

	K1 (n = 21)		K2 (n = 18)		K3 (n = 20)		K4 (n = 23)		K5 (n = 19)	
	**Male**	**Female**	**Male**	**Female**	**Male**	**Female**	**Male**	**Female**	**Male**	**Female**
**Sex**	12 (57%)	9 (43%)	5 (28%)	13 (72%)	6 (30%)	14(70%)	13 (57%)	10 (43%)	11 (58%)	8 (42%)
	**Mean**	**SD**	**Mean**	**SD**	**Mean**	**SD**	**Mean**	**SD**	**Mean**	**SD**
**Age, years**	24.81	6.51	23.39	3.70	23.95	4.98	25.18	5.04	23.05	3.64
**Perseverative thinking questionnaire (PTQ)**	20.38	13.16	12.72	10.52	13.25	9.18	16.35	11.48	17.11	13.94
**CNBTQ[a] (pre-task)**	2.71	0.72	2.57	1.14	2.62	0.90	2.61	1.08	2.44	0.92

[a]Challenging Negative Beliefs Task Questionnaire (CNBTQ) surveys participants' endorsement of the negative self-cognition statements (1 = Strongly Disagree; 7 = Strongly Agree) presented during the fMRI cognitive restructuring paradigm. This was completed before and after the MRI brain scan to assess changes in negative self-cognition endorsement after undergoing the cognitive restructuring task.
SD standard deviation.

Supplementary Table 9. List of negative self-cognition statements in the cognitive restructuring paradigm

1. I am incompetent in most things I do.
2. I don't measure up to others.
3. I am insignificant.
4. Other people are more competent than I am.
5. Even if I make an effort, I will fail.
6. It is unlikely that someone will ever be attracted to me.
7. I will always be rejected if people discover my flaws.
8. All things considered, I'm a failure.
9. I'm boring and uninteresting.
10. I'm not good enough to be loved.
11. People want to take advantage of me.
12. People want me to fail.
13. I am incapable of changing my life.
14. I can't do anything right.
15. I have little value as a person.
16. People will hurt me in order to get what they need.
17. My value depends on my body shape.
18. I'm only successful when I am at my ideal weight.
19. People will not accept me because of my body shape.
20. People think poorly of me because of my weight.
21. I won't be able to stop if I eat what I really want.
22. I have no control when it comes to food.
23. I can't be trusted around certain foods.
24. I must avoid eating certain foods to stay in control.

The abbreviated version of the cognitive restructuring paradigm presented to the replication sample included statements 1-16. The general negative self-cognition statements (items 1-16) were adapted from those reported in the cognitive behavioural therapy literature[7, 8]. Pathological cognitions related to food and body image (items 17-24) were adapted from the Eating Disorder Belief Questionnaire[9].

Supplementary Figures

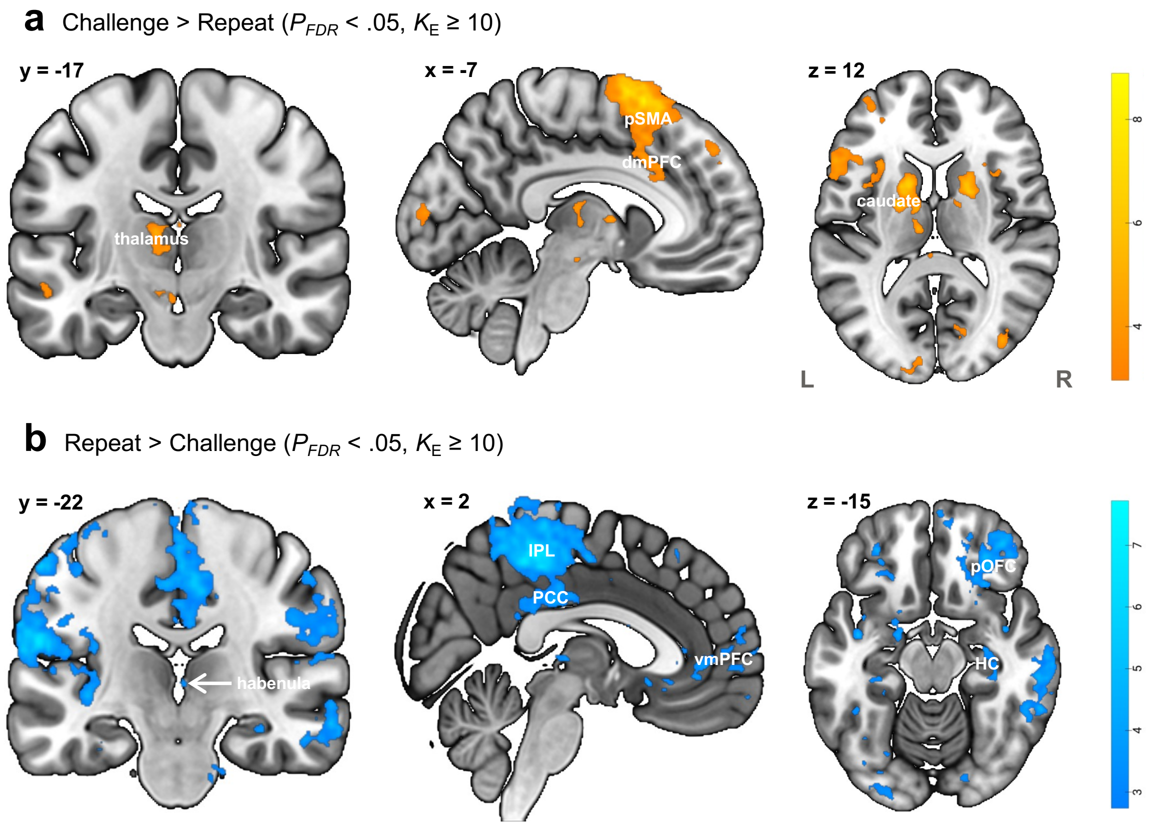

Supplementary Figure 1. Task-elicited neural activation in the discovery sample (*n* = 48)

a The first heatmap illustrates the general linear model results of the Challenge > Repeat contrast showing brain regions with increased activation during the cognitive restructuring of negative self-cognitions compared to repetition (P_{FDR}<0.05, K_E≥10). **b** Likewise, the second heatmap shows the results of the Repeat > Challenge contrast indicating brain regions that are significantly more activated during the repeating relative to the restructuring of negative self-cognitions (P_{FDR}<0.05, K_E≥10). Colour bar represents the t-value range (one sample t-test, one-tailed) for the corresponding contrast. Results are displayed on the MNI152 T1 0.5 mm template.

dmPFC dorsomedial prefrontal cortex, *HC* hippocampus, *IPL* inferior parietal lobule, *L* left, *PCC* posterior cingulate cortex, *pOFC* posterior orbitofrontal cortex, *pSMA* pre-supplementary motor area, *R* right, *vmPFC* ventromedial prefrontal cortex.

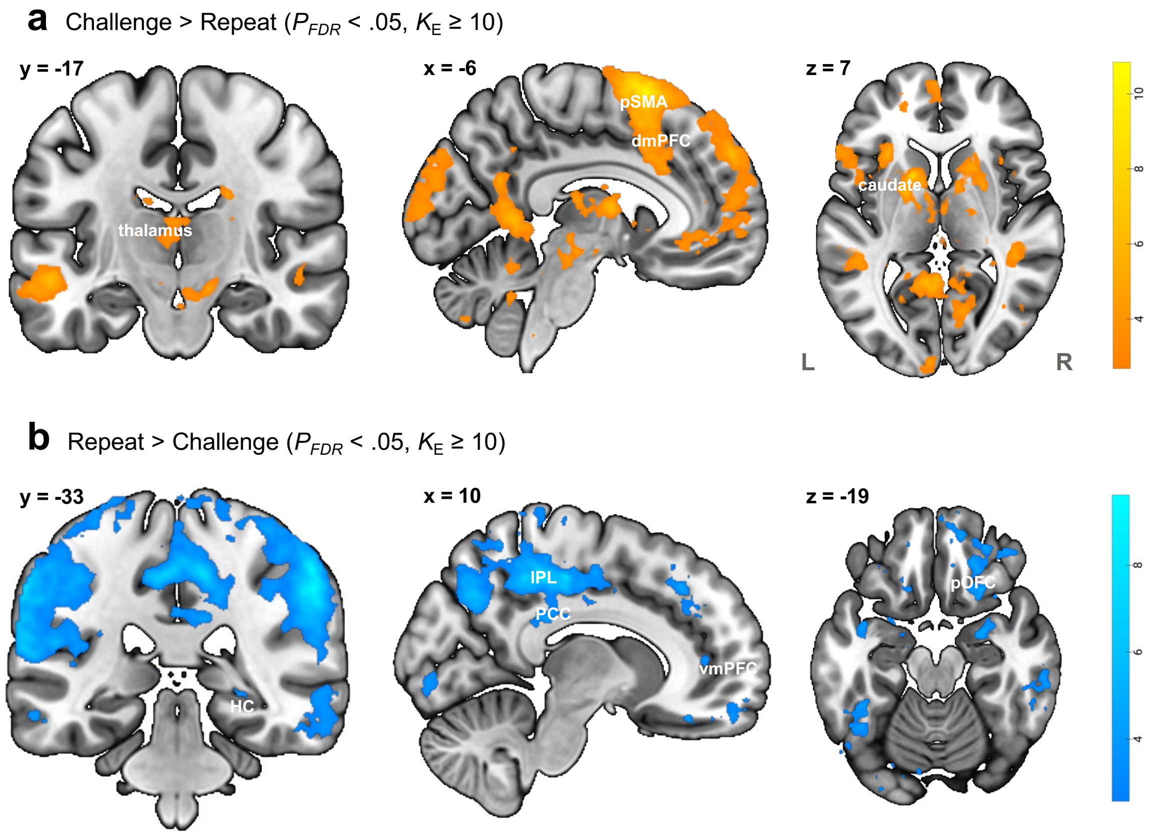

Supplementary Figure 2. Task-elicited neural activation in the replication sample (*n* = 65)

a The first heatmap illustrates the general linear model results of the Challenge > Repeat contrast showing brain regions with increased activation during the cognitive restructuring of negative self-cognitions compared to repetition (P_{FDR}<0.05, K_E≥10). **b** Likewise, the second heatmap shows the results of the Repeat > Challenge contrast indicating brain regions that are significantly more activated during the repeating relative to the restructuring of negative self-cognitions (P_{FDR}<0.05, K_E≥10). Colour bar represents the t-value range (one sample t-test, one-tailed) for the corresponding contrast. Results are displayed on the MNI152 T1 0.5 mm template.

dmPFC dorsomedial prefrontal cortex, *HC* hippocampus, *IPL* inferior parietal lobule, *L* left, *PCC* posterior cingulate cortex, *pOFC* posterior orbitofrontal cortex, *pSMA* pre-supplementary motor area, *R* right, *vmPFC* ventromedial prefrontal cortex.

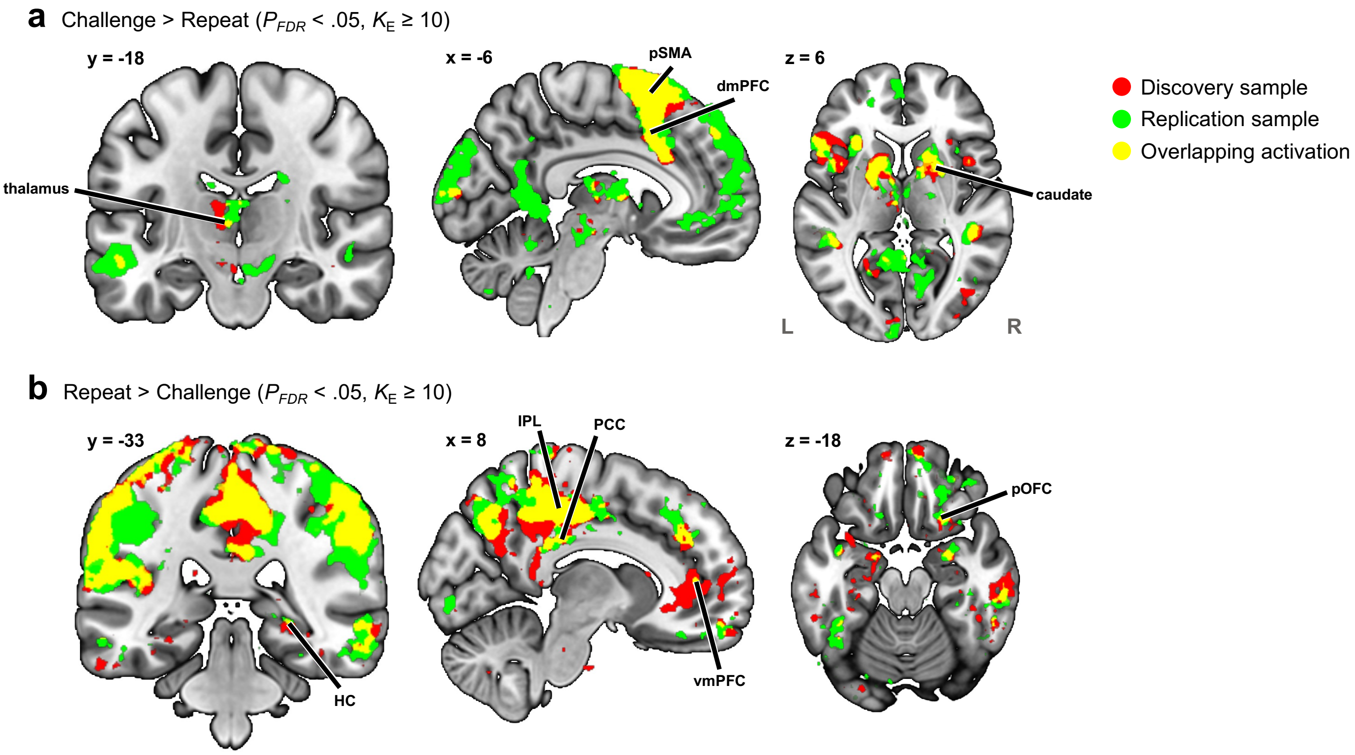

Supplementary Figure 3. Overlapping activation across the discovery and replication datasets

a The colour maps depict the general linear model results from the discovery and replication samples, respectively (P_{FDR}<0.05, K_E≥10), as well as their spatial overlap. The red colour map in the first row shows brain regions exhibiting increased activation during the restructuring of negative self-cognitions compared to repeating in the discovery sample, whereas the green colour map displays results from the same contrast in the replication sample. The yellow colour map illustrates the overlapping activation pattern across the two samples. Cognitive restructuring elicited heightened left-lateralised activation in frontostriatal cognitive control regions that are consistent across the two samples. **b** The colour map in the second row shows brain regions with increased activation during the repeating compared to the restructuring of negative self-cognitions in the discovery (red) and replication (green) samples (P_{FDR}<0.05, K_E≥10). The yellow colour map shows the extent to which brain activation patterns in the two samples overlap. During the repetition of negative self-cognitions, both samples had increased activation in the bilateral default mod network, alongside the right orbitofrontal cortex and the right hippocampus.

dmPFC dorsomedial prefrontal cortex, *HC* hippocampus, *IPL* inferior parietal lobule, *L* left, *PCC* posterior cingulate cortex, *pOFC* posterior orbitofrontal cortex, *pSMA* pre-supplementary motor area, *R* right, *vmPFC* ventromedial prefrontal cortex.

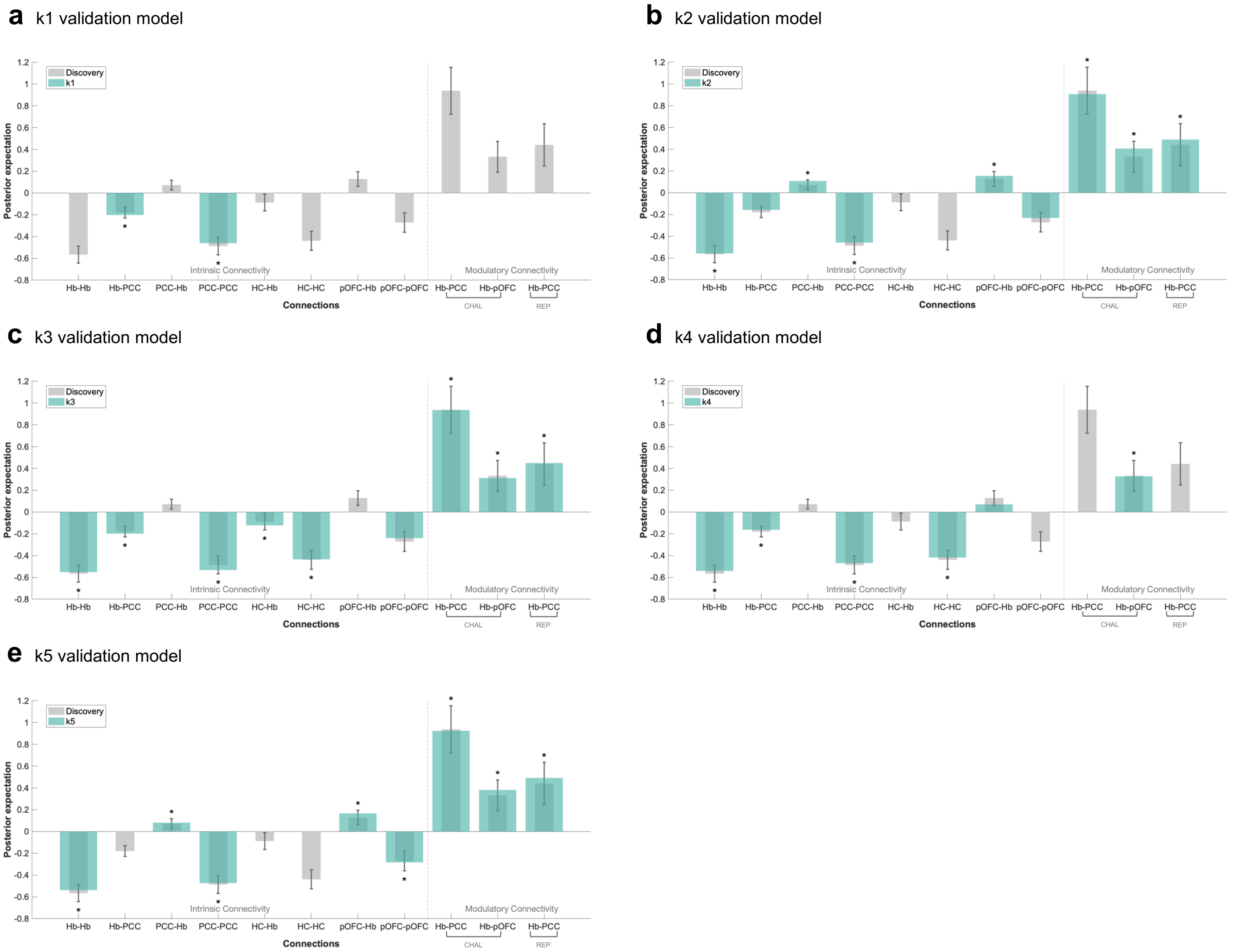

Supplementary Figure 4. Intrinsic and modulatory connectivity comparison of the discovery and 5-fold validation models

a-e The figure illustrates intrinsic and modulatory effective connectivity with a non-zero posterior probability of the discovery (grey) model superimposed with the posterior expectation of the 5-fold validation models (green) for comparison (k1-k5, n = 21/18/20/23/19). The bars represent the Bayesian model-averaged (BMA) connectivity strength estimates of the corresponding network connection in the models, with the whiskers representing their respective 95% confidence interval (CI) derived from the posterior covariance matrix of the discovery model (spm_plot_ci.m). An asterisk above the green bar highlights validation model connectivity that has an associated posterior probability >.95, indicating strong evidence for a group-level effect. Connectivity that is consistent across the models is identified based on the connectivity estimates in the validation model that fall within the 95% CI of the discovery model estimate and satisfy the posterior probability threshold (PP >.95). The positive modulatory effect of both the restructuring (CHAL) and repeating (REP) negative self-cognitions on the habenula-to-PCC connection met a posterior probability >.95 threshold in 3 out of 5 of the validation models. The positive modulatory effect of cognitive restructuring (CHAL) on the habenula-to-pOFC pathway met a posterior probability >.95 threshold in 4 out of 5 validation models.

CHAL challenge condition, *Hb* habenula, *HC* hippocampus, *PCC* posterior cingulate cortex, *pOFC* posterior orbitofrontal cortex, *REP* repeat condition.

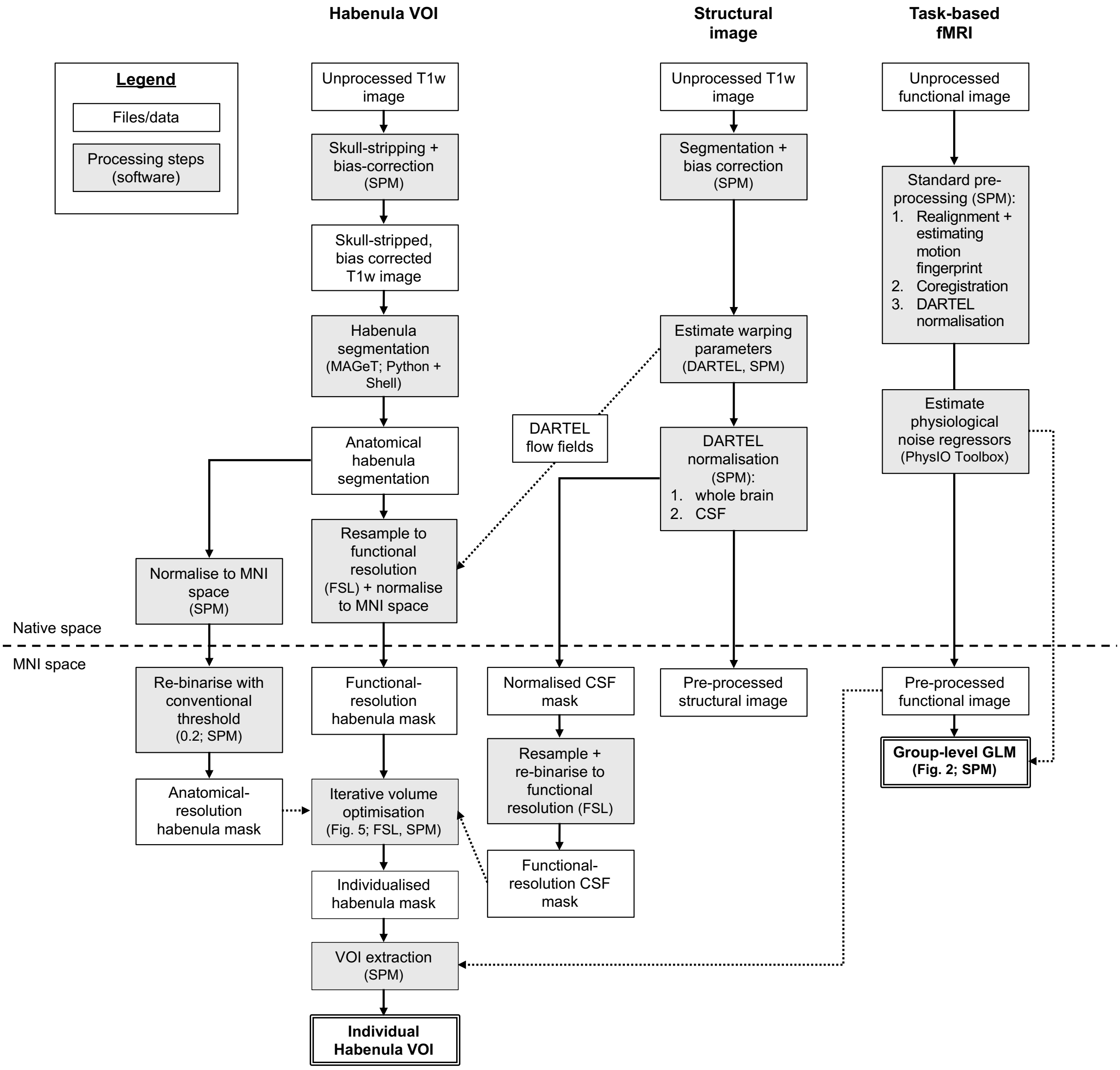

Supplementary Figure 5. Overview of the habenula VOI preparation pipeline

Anatomical and functional MRI images were acquired for the present study. Standard pre-processing was applied including spatial realignment, co-registration, segmentation, normalisation, motion[10] and physiological noise correction[2]. SPM12 and FSL were used during image processing. Pre-processed functional images in the MNI space were used for the GLM analysis to probe task-induced neural activation. Anatomical images in the native space were used to generate individualised habenula masks via the MAGeTbrain algorithm[11, 12]. DARTEL flow fields generated during anatomical image pre-processing were used to transform individualised habenula masks to the standard space. Normalised habenula and CSF masks were used as part of the iterative volume optimisation procedure when creating individualised habenula VOI in functional resolution for the dynamic causal model analysis.

CSF cerebrospinal fluid, *DARTEL* Diffeomorphic Anatomical Registration Through Exponentiated Lie Algebra, *fMRI* functional magnetic resonance imaging, *FSL* FMRIB Software Library, *GLM* general linear model, *MAGeT* Multiple Automatically Generated Templates brain segmentation, *MNI* Montreal Neurological Institute, *SPM* Statistical Parametric Mapping, *T1w* T1-weighted, *VOI* volume-of-interest.

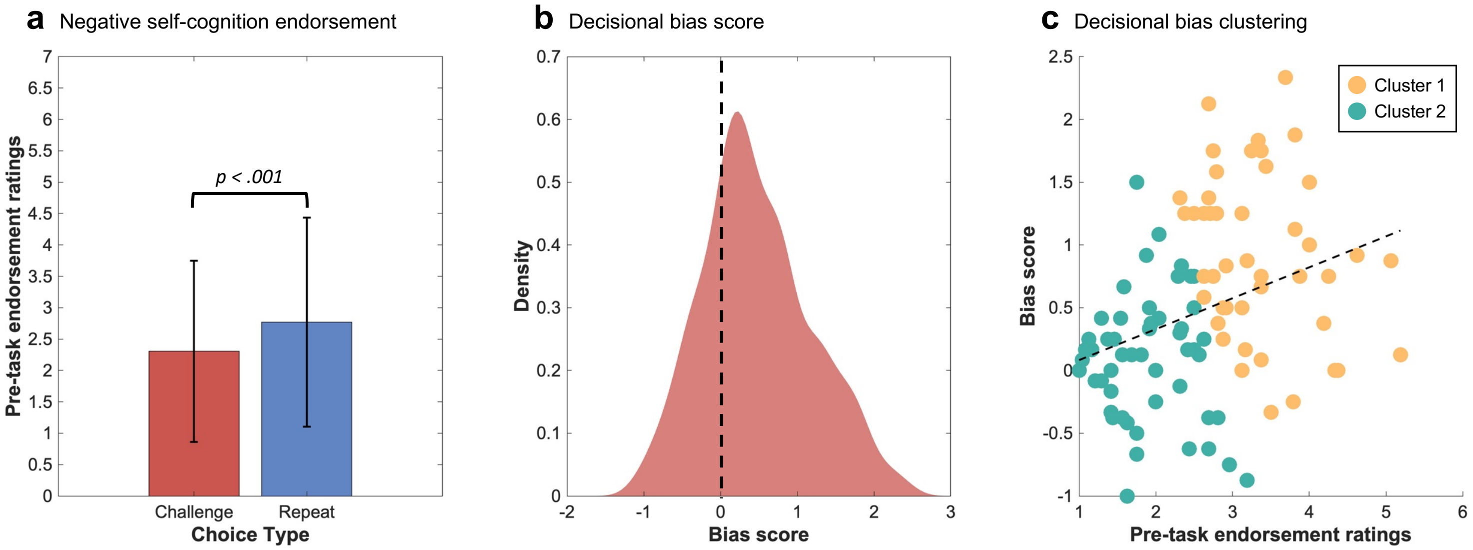

Supplementary Figure 6. Participant negative self-cognition endorsement and choice bias during the cognitive restructuring task

a The bar graph depicts the mean and standard deviation of participant endorsement ratings for negative self-cognition statements that were either restructured/challenged or repeated during the cognitive restructuring task. Mean endorsement scores were higher for repeated statements compared to challenged statements ($Mean_{difference}$ = -0.48, t_{98} = -6.70, $P_{bonf.\text{-}corrected}$ < .001, d = -0.67, 95% CI = [-0.62, -0.33]). **b** The distribution plot shows participant choice biases calculated as the difference in endorsement ratings between statements that were challenged or repeated. Higher scores indicate an increased tendency to repeat statements that were more highly endorsed. **c** The scatter plot depicts the association between participant choice biases during the cognitive restructuring task and pre-task negative self-cognition endorsement ratings. A significant positive association was identified between choice biases and endorsement ratings (r = .33, $P_{bonf.\text{-}corrected}$ < .001, 95% CI = [0.14, 0.50]), indicating that participants with an overall higher negative self-cognition endorsement rating were more likely to repeat the statements they more strongly endorsed. This was further tested using k-means clustering. The green cluster (n = 53) had significantly lower endorsement rating ($Mean_{difference}$ = -1.43, t_{97} = -11.31, $P_{bonf.\text{-}corrected}$ < .001, d = -2.28, 95% CI = [-1.69, -1.18]) and bias scores ($Mean_{difference}$ = -0.82, t_{97} = -7.02, $P_{bonf.\text{-}corrected}$ < .001, d = -1.41, 95% CI = [-1.05, -0.59]) compared to the orange cluster (n = 46).

Supplementary References

1. Nieto-Castanon A, Whitfield-Gabrieli S. *CONN functional connectivity toolbox: RRID SCR_009550 release 22* (2022).

2. Kasper L*, et al.* The PhysIO toolbox for modeling physiological noise in fMRI data. *J. Neurosci. Methods* **276**, 56-72 (2017).

3. Agathos J*, et al.* Differential engagement of the posterior cingulate cortex during cognitive restructuring of negative self- and social beliefs. *Soc. Cogn. Affect. Neurosci.* **18**, (2023).

4. Steward T*, et al.* A thalamo-centric neural signature for restructuring negative self-beliefs. *Mol. Psychiatry* **27**, 1611-1617 (2022).

5. Rolls ET, Huang C-C, Lin C-P, Feng J, Joliot M. Automated anatomical labelling atlas 3. *Neuroimage* **206**, 116189 (2020).

6. Zeidman P*, et al.* A guide to group effective connectivity analysis, Part 2: Second level analysis with PEB. *Neuroimage* **200**, 12-25 (2019).

7. Beck AT, Dozois DJ. Cognitive therapy: Current status and future directions. *Annu. Rev. Med.* **62**, 397-409 (2011).

8. Osmo F*, et al.* The Negative Core Beliefs Inventory: Development and psychometric properties. *J. Cogn. Psychother.* **32**, 67-84 (2018).

9. Cooper M, Cohen-Tovée E, Todd G, Wells A, Tovée M. The Eating Disorder Belief Questionnaire: Preliminary development. *Behav. Res. Ther.* **35**, 381-388 (1997).

10. Wilke M. An alternative approach towards assessing and accounting for individual motion in fMRI timeseries. *Neuroimage* **59**, 2062-2072 (2012).

11. Chakravarty MM*, et al.* Performing label-fusion-based segmentation using multiple automatically generated templates. *Hum. Brain Mapp.* **34**, 2635-2654 (2013).

12. Germann J*, et al.* Fully Automated Habenula Segmentation Provides Robust and Reliable Volume Estimation Across Large Magnetic Resonance Imaging Datasets, Suggesting Intriguing Developmental Trajectories in Psychiatric Disease. *Biol. Psychiatry Cogn. Neurosci. Neuroimaging* **5**, 923-929 (2020).
