# Supplementary material for: Habenula contributions to negative self-cognitions
Source: Nat Commun. 2025 May 7;16:4231. doi: 10.1038/s41467-025-59611-7 (PMC12059057; doi:10.1038/s41467-025-59611-7)
Supplement: Supplementary file 2 — Reporting Summary [file 41467_2025_59611_MOESM2_ESM.pdf]

nature portfolio

Corresponding author(s): Trevor Steward, PhD

Last updated by author(s): Apr 7, 2025

Reporting Summary

Nature Portfolio wishes to improve the reproducibility of the work that we publish. This form provides structure for consistency and transparency in reporting. For further information on Nature Portfolio policies, see our Editorial Policies and the Editorial Policy Checklist.

Statistics

For all statistical analyses, confirm that the following items are present in the figure legend, table legend, main text, or Methods section.

n/a	Confirmed	
☐	☒	The exact sample size (*n*) for each experimental group/condition, given as a discrete number and unit of measurement
☐	☒	A statement on whether measurements were taken from distinct samples or whether the same sample was measured repeatedly
☐	☒	The statistical test(s) used AND whether they are one- or two-sided<br>*Only common tests should be described solely by name; describe more complex techniques in the Methods section.*
☐	☒	A description of all covariates tested
☐	☒	A description of any assumptions or corrections, such as tests of normality and adjustment for multiple comparisons
☐	☒	A full description of the statistical parameters including central tendency (e.g. means) or other basic estimates (e.g. regression coefficient) AND variation (e.g. standard deviation) or associated estimates of uncertainty (e.g. confidence intervals)
☐	☒	For null hypothesis testing, the test statistic (e.g. *F*, *t*, *r*) with confidence intervals, effect sizes, degrees of freedom and *P* value noted<br>*Give P values as exact values whenever suitable.*
☐	☒	For Bayesian analysis, information on the choice of priors and Markov chain Monte Carlo settings
☒	☐	For hierarchical and complex designs, identification of the appropriate level for tests and full reporting of outcomes
☐	☒	Estimates of effect sizes (e.g. Cohen's *d*, Pearson's *r*), indicating how they were calculated

Our web collection on statistics for biologists contains articles on many of the points above.

Software and code

Policy information about availability of computer code

Data collection	E-Prime 3.0 software (Psychology Software Tools, Pittsburgh, PA) was used to present the cognitive restructuring paradigm. Microsoft PowerPoint 2021 was used to display the fixation cross during the resting-state scan.
Data analysis	MRI data pre-processing was conducted with SPM12 (v7771; Wellcome Trust Centre for Neuroimaging, London) in the Matlab 2023a environment (The MathWorks Inc., Natick, MA) and FMRIB Software Library (FSL) 6.0.6.5. PhysIO Toolbox (Kasper et al., 2017) was used for physiological noise correction. MAGeTbrain algorithm was used for habenula segmentation (Germann et al., 2021). Effective connectivity analysis was conducted using SPM12 and the codes are available at "https://github.com/pohankung/NegativeBeliefs_Habenula_DCM". Resting-state analysis was completed using the CONN toolbox (v22).

For manuscripts utilizing custom algorithms or software that are central to the research but not yet described in published literature, software must be made available to editors and reviewers. We strongly encourage code deposition in a community repository (e.g. GitHub). See the Nature Portfolio guidelines for submitting code & software for further information.

Data

Policy information about availability of data

All manuscripts must include a data availability statement. This statement should provide the following information, where applicable:

- Accession codes, unique identifiers, or web links for publicly available datasets
- A description of any restrictions on data availability
- For clinical datasets or third party data, please ensure that the statement adheres to our policy

Deidentified effective connectivity data that support the findings of this study are publicly available at "https://github.com/pohankung/NegativeBeliefs_Habenula_DCM". Source data for Fig. 3b and 4b are provided with the paper.

Research involving human participants, their data, or biological material

Policy information about studies with human participants or human data. See also policy information about sex, gender (identity/presentation), and sexual orientation and race, ethnicity and racism.

Reporting on sex and gender	The aim of the study was to characterise habenula connectivity underpinning negative self-cognition processing in the general population. Approximately equal numbers of males and females (sex) were recruited, and reported in Supplementary Table 1. Participants' sex was based on self-report of assigned-sex-at-birth and all participants provided informed consent for data sharing.
Reporting on race, ethnicity, or other socially relevant groupings	Participants' self-reported etho-cultural identity is reported in Supplementary Table 1.<br>- The discovery sample included 28 individuals identifying as Asian, 2 as Australian (non-Aboriginal or Torres Strait Islander), 6 as British/European, 1 as North American, 2 as Central/South American, 1 as Middle Eastern, and 8 identified with more than one ethno-cultural groups.<br>- The replication sample included 23 individuals identifying as Caucasian, 38 as Asian, 3 as Latinx, and 1 as Mixed.
Population characteristics	Both samples consisted of healthy adults recruited from the community. Age and sex summaries are detailed in Supplementary Table 1.
Recruitment	All participants were recruited via publicy available online advertisement and resided in the Greater Melbourne area with accessibility to the scanning facility, where this study was conducted. All participants were provided with a Plain Language Statement (PLS) outlining the study procedure. It is unlikely that participants who are uncomfortable with MRI scanning environments would volunteer to take part in this research. These may have minimally impacted the external validity of our obtained results.
Ethics oversight	All participants provided written informed consent for anonymised data sharing. This study was approved by the University of Melbourne Human Research Ethics Committee (HREC 22347, 2056265).

Note that full information on the approval of the study protocol must also be provided in the manuscript.

Field-specific reporting

Please select the one below that is the best fit for your research. If you are not sure, read the appropriate sections before making your selection.

☒ Life sciences ☐ Behavioural & social sciences ☐ Ecological, evolutionary & environmental sciences

For a reference copy of the document with all sections, see nature.com/documents/nr-reporting-summary-flat.pdf

Life sciences study design

All studies must disclose on these points even when the disclosure is negative.

Sample size	The current study consists of two tasked-based datasets (discovery: n = 48; replication: n = 65) and one resting-state dataset (n = 50). No statistical method was used to predetermine the sample size, but our sample sizes are comparable to that recommended for stable dynamic causal model and functional connectivity analysis at standard field-strength (3-tesla; Silchenko et al., 2023; Ma et al., 2024; Termenon et al., 2016). Note that these recommendations are based on resting-state data and task-based guidelines are lacking. With that said, our use of ultra-high field 7-Tesla MRI likely yields improved signal-to-noise ratio that enables sufficient statistical power at smaller sample sizes (Torrisi et al., 2017). Our results show reproducible effective connectivity estimates across the two independent samples and our resting-state analysis revealed consistent connectivity pattern as past reports (Ely et al., 2019; Torrisi et al., 2017).
Data exclusions	Nine and 18 participants were excluded from the discovery (n = 57) and replication (n = 83) samples from the neural activation analysis, respectively, due to technical errors in MRI acquisition (discovery: 1, replication: 3), participant not completing or incorrectly completing the fMRI paradigm (discovery: 7, replication: 5), and excessive head motion (discovery: 1, replication: 10). An additional 3 participants from the discovery sample and 9 from the replication sample were excluded from the task-based DCM analysis due to a failure to extract a complete set of VOIs.
Replication	The independent replication sample (n = 56) was used to assess the replicability of our habenula connectivity model based on the discovery sample (n = 45). Consistent modulatory effective connectivity was observed for the habenula-to-pOFC pathway during cognitive restructuring.

	This was further assessed via a randomised stratified 5-fold validation to evaluate the impact of individual variance. Four out of 5 validation models reproduced the modulatory effect of cognitive restructuring on the habenula-to-pOFC connection, supporting the replicability of our findings.
Randomization	All participants underwent the cognitive restructuring paradigm during MRI scanning, hence, no randomisation procedure was used for our discovery and replication DCM analysis. The aim of the study was to establish a model of normative habenula effective connectivity, no covariates were controlled.<br>Participants from both the discovery and replication samples were combined and randomised into 5 sub-samples for our 5-fold validation analysis using custom code available at https://github.com/pohankung/NegativeBeliefs_Habenula_DCM.
Blinding	No blinding was conducted since there were no group variables with respect to the connectivity or behavioural variables.

Reporting for specific materials, systems and methods

We require information from authors about some types of materials, experimental systems and methods used in many studies. Here, indicate whether each material, system or method listed is relevant to your study. If you are not sure if a list item applies to your research, read the appropriate section before selecting a response.

Materials & experimental systems

n/a	Involved in the study	
☒	☐	Antibodies
☒	☐	Eukaryotic cell lines
☒	☐	Palaeontology and archaeology
☒	☐	Animals and other organisms
☒	☐	Clinical data
☒	☐	Dual use research of concern
☒	☐	Plants

Methods

n/a	Involved in the study	
☒	☐	ChIP-seq
☒	☐	Flow cytometry
☐	☒	MRI-based neuroimaging

Plants

Seed stocks	N/A
Novel plant genotypes	N/A
Authentication	N/A

Magnetic resonance imaging

Experimental design

Design type	Task-based fMRI; block design; resting-state.
Design specifications	For the primary task-based data, participants underwent a cognitive restructuring paradigm consisting one run of 24 blocks (16 blocks for the replication sample). During each block, participants were presented with a negative self-cognition statement which they either restructured or repeated in their minds. The secondary resting-state data comprised one run lasting just over 5 minutes, where participants were instructed to focus on a fixation cross while remaining relaxed and awake.
Behavioral performance measures	Participants indicated their choice to restructure or repeat each statement using a MRI-compatible button box. This information was considered when analysing the change in negative self-cognition endorsement from before to after the MRI task. A set of self-report questionnaires were administered outside of the scanner, including participants' negative self-cognition endorsement (CNBTQ) and proclivity for perseverative thinking (PTQ).

Acquisition

Imaging type(s)	Functional, structural
Field strength	7-Tesla
Sequence & imaging parameters	Discovery dataset

- Functional (task): multi-band (factor = 6) and GRAPPA (R = 2) accelerated GE-EPI sequence in the steady state (TR = 800 ms; TE = 22.2 ms; pulse angle = 45 degrees; field of view = 20.8 cm; acquisition matrix = 130 x 130-pixel; slice thickness = 1.6 mm, no gap)
- Structural: multi-echo Magnetization Prepared 2 Rapid Acquisition Gradient Echoes sequence (ME-MP2RAGE; 224 interleaved axial slices; TR = 4500 ms; TE = 2.21/4.21/6.15/8.14 ms; inversion time = 700/2700 ms; flip angle = 6/7 degrees; field of view = 24 cm; acquisition matrix = 320 x 320-pixel; slice thickness = 0.75 mm, no gap)
- Resting state: multi-band (factor = 6) and GRAPPA (R = 2) accelerated GE-EPI sequence in the steady state (TR = 800 ms; TE = 22.2 ms; pulse angle = 45 degrees; field of view = 20.8 cm; acquisition matrix = 130 x 130-pixel; slice thickness = 1.6 mm, no gap; 84 interleaved axial slices)

Replication dataset
- Functional (task): same as the Discovery dataset
- Structural: single-echo MP2RAGE sequence (224 interleaved sagittal slices; TR = 5000 ms; TE = 2.04 ms; inversion time = 700/2700 ms; flip angle = 4/5 degrees; field of view = 24 cm; acquisition matrix = 320 x 320-pixel; slice thickness = 0.75 mm, no gap)

Area of acquisition: Whole-brain scan.

Diffusion MRI ☐ Used ☒ Not used

Preprocessing

Preprocessing software: Imaging data was pre-processed with SPM12 (v7771; Wellcome Trust Centre for Neuroimaging, London) within the MATLAB 2023a environment (The MathWorks Inc., Natick, MA). Functional images were realigned to the mean image to correct for movement during the scan, and all images were resampled using 4th Degree B-Spline interpolation. Functional images were spatially normalised with the DARTEL flow fields and smoothed with a 2 mm^3 full width at half maximum (FWHM) Gaussian kernel. Details for resting-state pre-processing is reported in the Supplementary Information.

Normalization: Functional EPI were normalised to the MNI template using the unified segmentation plus DARTEL approach in SPM12

Normalization template: ICBM152

Noise and artifact removal: Motion artifacts were accounted for using the 6 realignment parameters produced during image pre-processing. Cardiac and respiratory recordings were modelled using the PhysIO toolbox (Kasper et al., 2017). Motion artifacts, and 24 physiological noise regressors (i.e., 6 cardiac, 8 respiratory, 4 cardiac × respiratory regressors, 1 RRF, 1 CRF, top principal components and mean time series of the CSF and WM) were included in the GLM analysis as nuisance regressors. Low-frequency fluctuation was high-pass filtered at 128Hz. The FAST method was used to estimate temporal autocorrelation resulting from our sub-second TR. Details for resting-state noise removal is reported in the Supplementary Information.

Volume censoring: N/A

Statistical modeling & inference

Model type and settings: Dynamic causal model and Parametric empirical Bayes were used to estimate habenula effective connectivity following published guidelines on task-based group-effect analysis (Zeidman et al., 2019). Full model specification of the initial mass-univariate random effects neural activation analysis is reported in the Methods section.

Effect(s) tested: Strength of effective connectivity is measured by the DCM's posterior expectation per connection. Variance is captured by the posterior covariance term. For the neural activation analysis, one-sample t-test (one-tailed) was used to assess changes in neural activation patterns across the task conditions.

Specify type of analysis: ☒ Whole brain ☐ ROI-based ☐ Both

Statistic type for inference (See Eklund et al. 2016): Statistical threshold for the effective connectivity analysis is determined by the posterior probability associated with each connectivity parameter estimates (Pp > .95 suggesting strong evidence for meaningful group effect). Significance testing for the initial neural activation analysis was based on the test statistics and p-value of the one-sample t-test ($p < .05$; one-tailed; FDR-corrected; cluster extent threshold = 10).

Correction: False discovery rate (FDR) corrected p-value was used to account for multiple comparisons during the neural activation analysis.

Models & analysis

n/a	Involved in the study	
☐	☒	Functional and/or effective connectivity
☒	☐	Graph analysis
☒	☐	Multivariate modeling or predictive analysis

Functional and/or effective connectivity: Effective connectivity was estimated using DCM and PEB per published guidelines (Zeidman et al., 2019). Functional connectivity between the habenula seed and whole-brain voxels were represented as Fisher-transformed bivariate Pearson correlation coefficients using a weighted GLM on the subject level For the

entire sample, whole-brain connectivity of the habenula was evaluated at each voxel using multivariate parametric statistics with random-effects across subjects. Results were thresholded and reported at whole-brain corrected p < .05 (FDR-corrected), KE ≥ 10 voxels.
